# Subacute Sternal Mass in Childhood

**DOI:** 10.5334/jbsr.1715

**Published:** 2019-01-08

**Authors:** Laurence Verhaeghe, Murielle Herman

**Affiliations:** 1 KU Leuven, BE; 2 AZ Delta, BE

**Keywords:** Sternal mass, Pseudotumor, Ultrasound, Pediatrics

## Case

**Figure 1:**
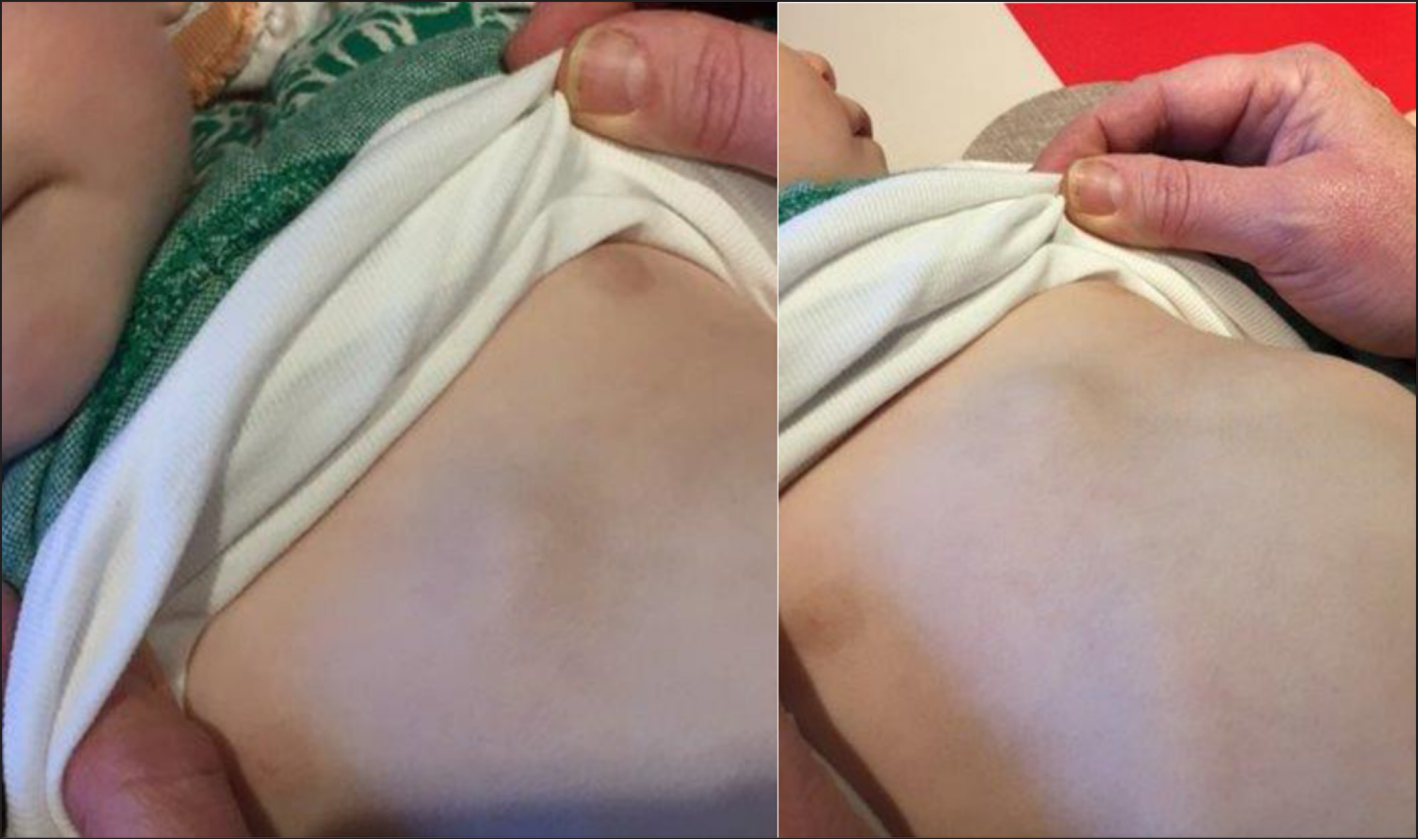
Clinical examination showed a sternal mass without any discoloration of the skin.

**Figure 2:**
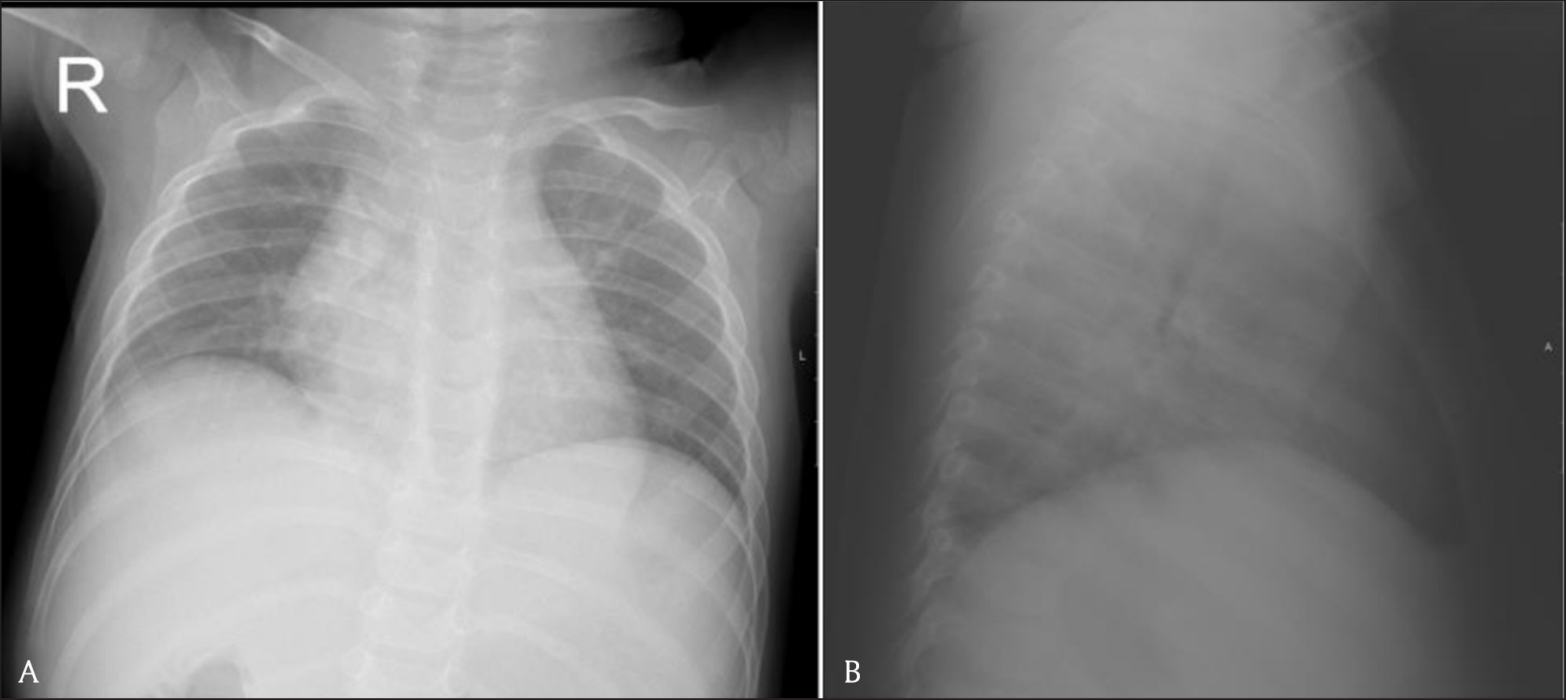
**A.** Peribronchial accentuation. **B.** Presternal mass without periostal reaction.

**Figure 3:**
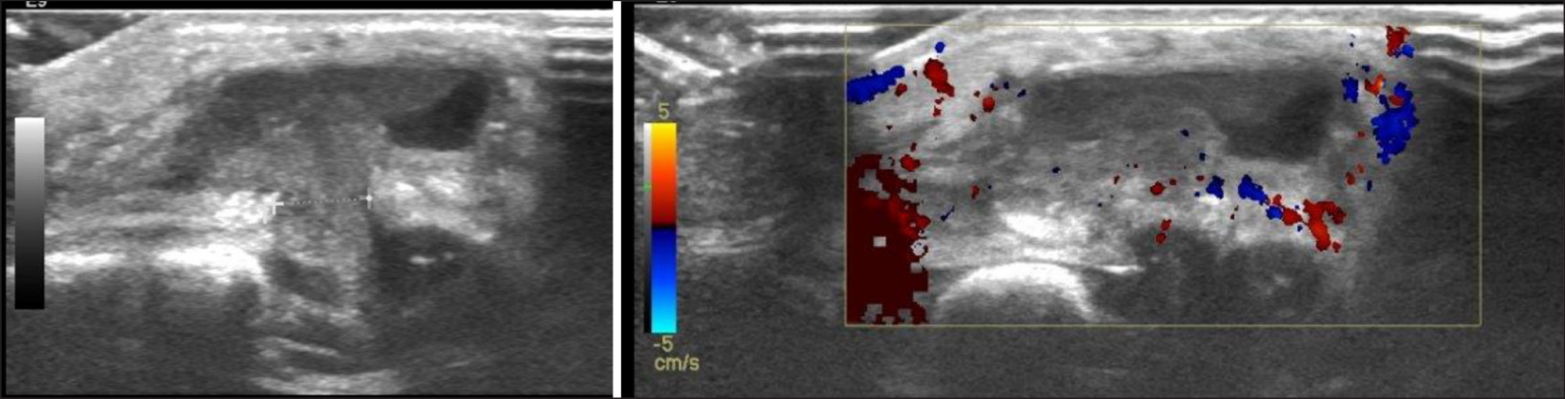
Ultrasonography shows a sharp defined lesion with a neck and presternal component (dumbbell sign) and is mainly hypo-echoic in comparison to the subcutaneous fat tissue. There is no internal vascularisation.

A nine-month-old girl presented to the pediatrician with signs of bronchitis. A few weeks earlier the parents had also noticed a sternal swelling, which was located paramedian right to the xiphoid process. On clinical examination (Figure 1) the swelling was painless, nontender, and showed neither rubor nor calor. X-ray (Figure 2a) showed peribronchial accentuation compatible with the diagnosis of (peri)bronchitis. On the lateral view (Figure 2b) a presternal tissue swelling without any periosteal reaction of the sternum was noticed. Ultrasonography (Figure 3) showed a soft tissue swelling which was composed of a retrosternal component, a neck between the sternum and the cartilage of the rib, and a presternal component. The lesion was sharply defined, inhomogeneous but mostly hypoechoic compared to the subcutaneous fat tissue, and showed no internal vascularization. There was no invasion of the surrounding tissues nor was there a connection to the skin. The absence of local/systemic inflammation as well as the absence of aggressive behavior led to a wait-and-see approach. On a follow-up ultrasound one week later, shrinkage of the mass was noticed. Taking into account the asymptomatic presentation of the lesion, the typical ‘dumbbell sign’ on ultrasound, and the spontaneous resolution, the diagnosis of a self-limiting sternal tumor of childhood (SELSTOC) was made.

## Discussion

Rapidly growing sternal tumors in children aren’t frequently encountered, but when they occur they raise a lot of concern often leading to invasive diagnostic procedures. To prevent unnecessary biopsies, it is important to be familiar with the entity of the self-limiting sternal tumors of childhood (SELSTOC). These benign processes are asymptomatic and don’t show any sign of local or systemic inflammation (in contrast to osteomyelitis, which is the differential diagnosis). On ultrasound they show a typical ‘dumbbell sign’ with a retrosternal component, a neck between the sternum and the cartilage of the adjacent rib, and a presternal component. They are sharply defined, are hypo-echoic compared to the surrounding subcutaneous fat, and can be surrounded by reactive fluid. There is no internal vascularization. No invasion of the surrounding bone or muscles nor a connection to the skin is seen. The asymptomatic presentation, the absence of aggressive behavior, together with the typical appearance on ultrasonography justify a wait-and-see approach, as these lesions consist of herniated mediastinal fat. In the case mentioned above, the bronchitis-related coughing led to a raised intrathoracic pressure, which was the underlying cause of the herniation of mediastinal fat. Ilivitzki et al. [1] suggest a first follow-up ultrasonography within 2–3 weeks and another one month later. Having confirmed the benign nature of the lesion, the interval between follow-up studies can widen.
